# Prospective multicentric validation of a novel prediction model for paroxysmal atrial fibrillation

**DOI:** 10.1007/s00392-020-01773-z

**Published:** 2020-11-19

**Authors:** Constanze Schmidt, Sebastian Benda, Patricia Kraft, Felix Wiedmann, Sven Pleger, Antonius Büscher, Dierk Thomas, Rolf Wachter, Christian Schmid, Roland Eils, Hugo A. Katus, Stefan M. Kallenberger

**Affiliations:** 1 Department of Cardiology, University Hospital Heidelberg, University of Heidelberg, Im Neuenheimer Feld 410, 69120 Heidelberg, Germany; 2 DZHK (German Center for Cardiovascular Research), Partner Site Heidelberg/Mannheim, University of Heidelberg, Im Neuenheimer Feld 410, 69120 Heidelberg, Germany; 3 Kardiologen Am Brückenkopf, Cardiology Practice, Brückenkopfstraße 1/2, 69120 Heidelberg, Germany; 4 Clinic and Policlinic for Cardiology, University Hospital Leipzig, Liebigstraße 18, 04103 Leipzig, Germany; 5 Clinic for Cardiology and Pneumology, University Medicine Göttingen, 37099 Göttingen, Germany; 6 Department of Internal Medicine, GPR Klinikum Rüsselsheim, August-Bebel-Straße 59, 65428 Rüsselsheim am Main, Germany; 7 Digital Health Center, Berlin Institute of Health (BIH) and Charité, Anna-Louisa-Karsch-Straße 2, 10178 Berlin, Germany; 8 Division of Theoretical Bioinformatics, German Cancer Research Center (DKFZ), Im Neuenheimer Feld 267, 69120 Heidelberg, Germany

**Keywords:** Paroxysmal atrial fibrillation, Atrial fibrillation detection, AF prediction model, ECHO-AF scores, Stroke prevention, Systems medicine

## Abstract

**Background:**

The early recognition of paroxysmal atrial fibrillation (pAF) is a major clinical challenge for preventing thromboembolic events. In this prospective and multicentric study we evaluated prediction scores for the presence of pAF, calculated from non-invasive medical history and echocardiographic parameters, in patients with unknown AF status.

**Methods:**

The 12-parameter score with parameters age, LA diameter, aortic root diameter, LV,ESD, TDI Aʹ, heart frequency, sleep apnea, hyperlipidemia, type II diabetes, smoker, ß-blocker, catheter ablation, and the 4-parameter score with parameters age, LA diameter, aortic root diameter and TDI A’ were tested. Presence of pAF was verified by continuous electrocardiogram (ECG) monitoring for up to 21 days in 305 patients.

**Results:**

The 12-parameter score correctly predicted pAF in all 34 patients, in which pAF was newly detected by ECG monitoring. The 12- and 4-parameter scores showed sensitivities of 100% and 82% (95%-CI 65%, 93%), specificities of 75% (95%-CI 70%, 80%) and 67% (95%-CI 61%, 73%), and areas under the receiver operating characteristic (ROC) curves of 0.84 (95%-CI 0.80, 0.88) and 0.81 (95%-CI 0.74, 0.87). Furthermore, properties of AF episodes and durations of ECG monitoring necessary to detect pAF were analysed.

**Conclusions:**

The prediction scores adequately detected pAF using variables readily available during routine cardiac assessment and echocardiography. The model scores, denoted as ECHO-AF scores, represent simple, highly sensitive and non-invasive tools for detecting pAF that can be easily implemented in the clinical practice and might serve as screening test to initiate further diagnostic investigations for validating the presence of pAF.

**Graphic abstract:**

Prospective validation of a novel prediction model for paroxysmal atrial fibrillation based on echocardiography and medical history parameters by long-term Holter ECG 
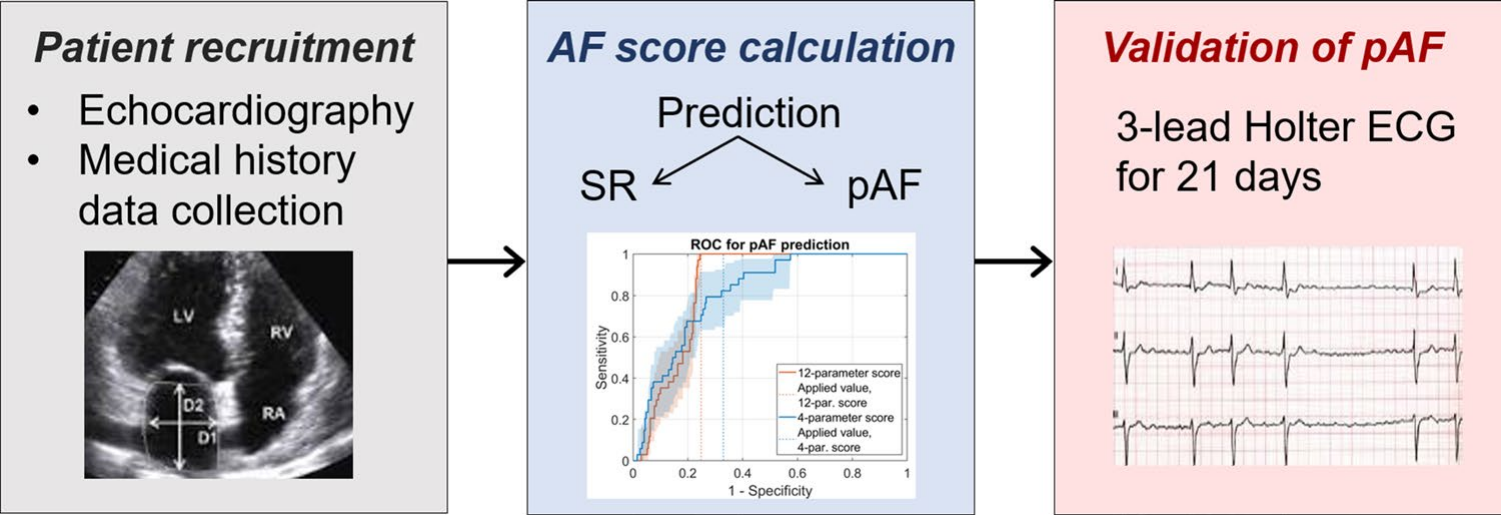

**Electronic supplementary material:**

The online version of this article (10.1007/s00392-020-01773-z) contains supplementary material, which is available to authorized users.

## Introduction

Atrial fibrillation (AF) is the most frequent arrhythmia but often remains unrecognized. Early detection is highly relevant to reducing stroke, heart failure and mortality but represents a major clinical challenge [1]. Developing new non-invasive methods for early diagnosis of paroxysmal AF (pAF) represents an important task to improve prevention of adverse effects of AF such as stroke, heart failure or cardiomyopathy [2, 3].

As part of the Apple Heart Study and the Huawei Heart Study, the use of smart watches for AF detection was evaluated in several hundred thousand subjects [4, 5]. The studies, however, were conducted in unselected populations. Therefore, AF was only detected in 0.5% or 0.2% of the study population. Population-level screening for AF bears the risk of large numbers of false-positive results and might cause unnecessary additional investigations or treatments [6]. Therefore, screening investigations are particularly valuable in patient groups with increased risk for AF [6]. Several models were established to predict the risk for developing AF in the next 5 or 10 years based on clinical data and blood biochemistry measures using Cox regression and procedures for selecting predictive parameters [7–12]. Scores developed within the CHARGE-AF consortium were challenged in independent studies based on electronic medical record databases and further developed using machine learning methods [12, 13].

It is well established that echocardiography is informative about the hemodynamic and mechanical cardiac functions. Several echocardiographic measures are associated with an increased risk for developing AF or AF-dependent complications such as left atrial (LA) enlargement, an increase of the left ventricular (LV) wall thickness or reduced end-diastolic to end-systolic fractional shortening of the LV [14–16]. Screening for pAF in patients undergoing echocardiography is reasonable because the prevalence of pAF in this patient collective is high. In the echocardiographic laboratory of the Department of Cardiology at Heidelberg University (Heidelberg, Germany), a prevalence of about 16% was observed [17]. Therefore, AF screening in this patient population is appropriate.

Recently, we systematically evaluated the predictive value of various echocardiographic parameters for AF and developed mathematical models and scores for predicting the presence of paroxysmal AF (pAF) using medical history and echocardiographic parameters that can be easily implemented in routine clinical practice [17]. The derivation cohort contained 47 clinical and echocardiographic parameters from 1000 patients. The optimal score variant includes 12 echocardiographic and medical history parameters that are most predictive for classifying between pAF and sinus rhythm (SR). To further simplify the clinical application, a reduced score with four parameters, age, LA diameter, tissue Doppler imaging velocity during atrial contraction (TDI, A’), and aortic root diameter, was developed.

In this prospective and multicentric study, we tested the pAF prediction scores in 305 patients with unknown AF status by continuous ECG monitoring over a period of up to 21 days. Thereby, we could validate the developed prediction scores, subsequently termed ‘ECHO-AF’ scores, as non-invasive tool for detecting pAF that can be easily implemented in clinical practice and might serve as a screening test to initiate further diagnostic investigations for validating the presence of pAF.

## Methods

### Study population

Echocardiographic and additional clinical data of 305 patients without diagnosis of AF or atrial flutter (50.2% males, 49.8% females) who were interested in undergoing a screening investigation for AF were included in this study between May 2016 and November 2019 at the Department of Cardiology and the Department of Neurology of the University Hospital of Heidelberg (Germany), and four cardiology practices in Germany (Heidelberg, Lüneburg, Essen). Patient data were collected in a de-identified manner. Based on the observed prevalence of pAF in our echocardiographic laboratory, we estimated that about 300 subjects were necessary to estimate sensitivity values with confidence intervals of ± 10% [18, 19]. The study protocol was approved by the ethics committee of the University of Heidelberg (Germany, Medical Faculty Heidelberg, S-491/2015). Written informed consent was obtained from all patients, and the study was conducted in accordance with the Declaration of Helsinki.

Collected score parameters consisted of medical history parameters (age, smoker, heart frequency, sleep apnea, hyperlipidemia, type 2 diabetes mellitus, catheter ablation), medication (beta-blocker), and echocardiographic parameters (aortic root diameter, left atrial diameter, left ventricular end-systolic diameter, TDI A’ velocity). Catheter interventions were ablations of accessory pathways or atrioventricular nodal reentry tachycardia. Patients with a history of atrial fibrillation or atrial flutter ablation were excluded.

### Echocardiography and long-term Holter ECG

Transthoracic echocardiography examinations were performed on commercially available ultrasound systems (GE Healthcare, Philips, Sony). Images included parasternal, apical and subxiphoidal views using 1.5–4.0 MHz phase-array transducers. All examinations were performed with 2D echocardiography for anatomic imaging and Doppler echocardiography for assessment of velocities. Left atrial size was determined as the maximal distance between the posterior aortic root wall and the posterior left atrial wall at the end of the systole. Aortic root and left ventricular end-systolic diameters (LV, ESD) were obtained in the parasternal long axis view. The TDI A’ velocity was measured in the apical four-chamber view. Patients carried 3-lead 7-day Holter ECG devices (Mortara Instruments) for up to 3 weeks. A few patients carried the device for 4 weeks. ECG recordings were evaluated by a cardiologist blinded to the score parameters and the calculated score values. AF was diagnosed in case AF episodes longer than 20 s were documented.

### Statistical methods

Continuous variables between SR and pAF groups were compared using one-way analysis of variance (ANOVA). Standard deviations are indicated by plus–minus signs (Table 1). Categorical variables between groups were compared with two-tailed Fisher exact test. Predictive scores were previously derived from logistic regression models calibrated with 47 parameters recorded in 1000 patients of the Department of Cardiology at Heidelberg University (Heidelberg, Germany) [17]. Of these 47 parameters, the most predictive 12 parameters were identified by sequential feature selection and likelihood-ratio testing. Scores were scaled between 0 and 100. The 12-parameter score is given by Eq. (1), and the 4-parameter score by Eq. (2):

1 $$\begin{aligned} L_{{12}} & = - 17.07 + 0.3359 \times \frac{{{\text{age}}}}{{\text{y}}} + 0.8700 \times \frac{{{\text{Ao,root}}}}{{{\text{mm}}}} + 0.7512 \times \frac{{{\text{LA}}}}{{{\text{mm}}}} - 0.3331 \times \frac{{{\text{LV,ESD}}}}{{{\text{mm}}}} \\ & - 1.570 \times \frac{{{\text{TDI,A}}^{'} }}{{{\text{cm}}/{\text{s}}}} + 0.1527 \times \frac{{{\text{HF}}}}{{1/\min }} + 10.98 \times {\text{sleep}}\;{\text{apnea}} + 4.172 \times {\text{hyperlipidemia}} - 0.1995 \times {\text{type}}\;{\text{II}}\;{\text{diabetes}} \\ & + 0.7565 \times {\text{smoker}} + 5.307 \times \beta \text{-} {\text{blocker}} + 21.39 \times {\text{catheter}}\;{\text{ablation}}. \\ \end{aligned}$$

2 $$L_{4} = - 22.96 + 0.4997 \times \frac{{{\text{age}}}}{{\text{y}}} + 0.9188 \times \frac{{{\text{Ao,root}}}}{{{\text{mm}}}} + 0.9459 \times \frac{{{\text{LA}}}}{{{\text{mm}}}} - 1.583 \times \frac{{{\text{TDI}},{\text{A}}^{'} }}{{{\text{cm}}/{\text{s}}}}.$$

**Table 1:** Comparison of score parameters between patient groups

	SR (*n* = 271)	pAF *(n* = 34)
Parameters		
Age (years)	58.7 ± 18.6	69.3 ± 12.5**
Aortic root (mm)	28.8 ± 6.3	34.3 ± 4.5***
LA, (mm)	34.4 ± 7.4	40.4 ± 5.5***
LV ESD (mm)	44.3 ± 12.6	34.2 ± 10.1***
Sleep apnea [*n* (%)]	28 (10)	1(3)
Hyperlipidemia [*n* (%)]	89 (33)	14(41)
Diabetes mellitus [*n* (%)]	38 (14)	7 (21)
Smoker [*n* (%)]	62 (23)	2 (6)*
Beta-blocker [*n* (%)]	110 (41)	17 (50)
Catheter ablation [*n* (%)]	18 (7)	6 (18)*
TDI A’ (cm/s)	8.2 ± 3.5	7.0 ± 4.2
Heart rate (1/min)	73.8 ± 12.6	76.8 ± 12.0

For continuous parameters, means and standard deviations are given, for categorical parameters with two levels, total counts and percentages are indicated

*LA* left atrium, *LV* ESD, left ventricular end-systolic diameter, *TDI* A’, tissue Doppler imaging, late diastolic velocity of mitral annulus

*p < 0.05, **p < 0.01, ***p < 0.001 versus SR from ANOVA for continuous variables and from Fisher exact test for categorical variables

In Eqs. (1) and (2), variables (age; Ao,root, aortic root diameter; LA, LA diameter; LV,ESD, LV end-systolic diameter; TDI,Aʹ, tissue Doppler imaging, late diastolic velocity of mitral annulus; *HF*, heart frequency) are divided by their units (y, years; mm, millimetres; cm/s, centimetres per second; 1/min, per minute). Categorical variables (sleep apnea; hyperlipidemia; type II diabetes; smoker; ß-blocker, ß-blocker intake; catheter ablation, status after catheter ablation) are set to 1 or 0 in case of their presence or absence. Using the 12-parameter score, presence of pAF was predicted in case of $L_{12} \ge 58.35$, and using the 4-parameter score, pAF was predicted in case of $L_{4} \ge 63.32$. An online calculator was created to simplify application of the scores [20].

For comparison, we assessed the predictive performance of logistic regression models containing only subsets of variables that are part of the 12-parameter score. We tested the following reduced models: (1) a model, reduced by all echocardiographic parameters, with parameters age, heart frequency, sleep apnea, hyperlipidemia, type II diabetes, smoker, beta-blocker, catheter ablation, (2) a model containing the parameters *age*, *gender*, *BMI*, and (3) a model containing only the parameter age. Parameters of these reduced models were obtained from calibration of logistic regression models based on the dataset that was previously used to establish the 4-parameter and 12-parameter scores [17]. ROC curves of these models were obtained as previously described using 100-fold cross-validation [17]. We further compared the predictive performance of our scores with two previous prediction scores, the HAVOC and the ACTEL scores [21, 22]. These two scores were developed to predict AF in patients with cryptogenic stroke or transient ischemic attack (TIA) and use non-invasive clinical parameters as well. In these scores, 95% confidence intervals were estimated by bootstrapping with *n* = 1000 samples.

To assess classification performance, area under the curve (AUC) values, sensitivities, specificities and precisions were analysed. We estimated 95% confidence intervals for sensitivity, specificity, precision and AUC values by bootstrapping with *n* = 1000 samples. All analyses were performed based on pre-implemented functions and custom scripts in MATLAB (MathWorks).

## Results

### Cohort characteristics and study outcomes

**Fig. 1:**
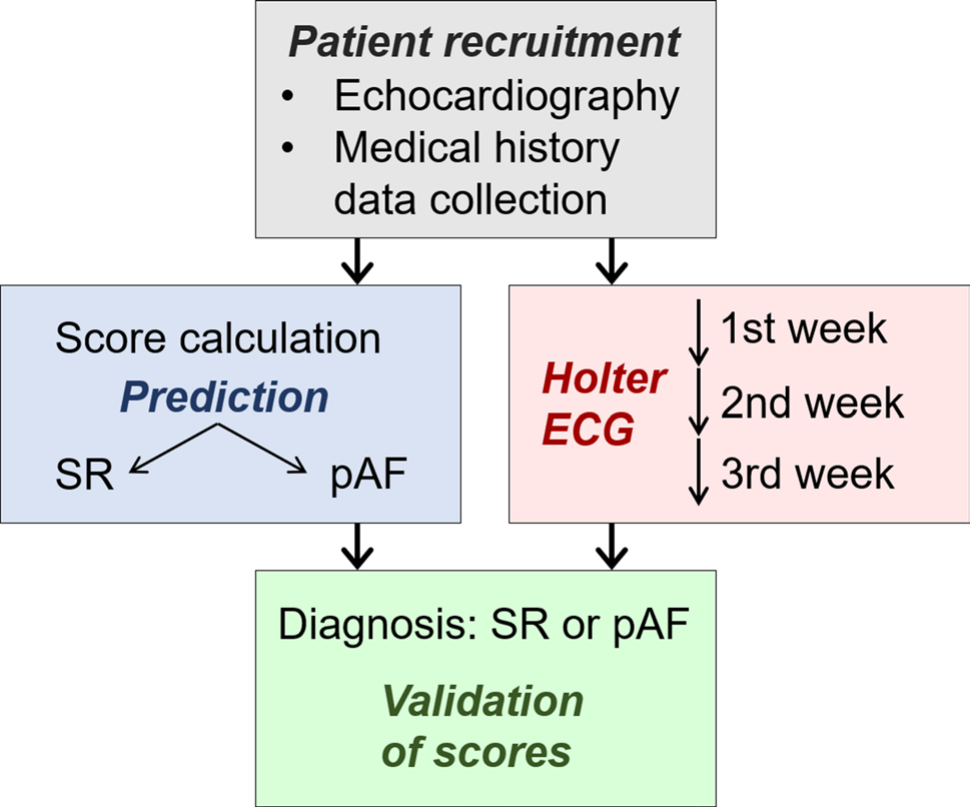
Design of the multicentric prospective study to evaluate the developed pAF prediction scores. In *n* = 305 patients, an echocardiographic investigation was conducted and medical history parameters were collected. Scores were calculated to predict the presence of SR or pAF. Holter ECG monitors were carried for up to 3 weeks and evaluated by medical personnel blinded to predictions based on scores. Diagnoses of pAF based on Holter ECG recordings were used to validate predictions from scores

Patients were included in the study at the Department of Cardiology and the Department of Neurology at Heidelberg University as well as four cardiology practices (age between 18 and 93 years, median age: 63, 50.2% males, 49.8% females; Supplementary Fig. S1). Patients undergoing an ambulatory echocardiographic investigation as well as healthy volunteers were offered participation in the study (Fig. 1). Patients were asked to wear Holter ECG devices for up to 3 weeks. Patients carried Holter ECG devices on average for 12.4 days (newly diagnosed pAF patients: 12.1 ± 7.8 days; SR patients: 12.4 ± 7.0 days; Supplementary Fig. S2). Table 1 gives an overview of the score parameters in SR and pAF patients. In addition, we compared measurements of a selection of biomarkers that were available for our patient collective (GFR, creatinine, Troponin-T hs, NT-proBNP). These parameters were previously associated with an increased risk for AF [11, 23, 24]. In our study sample, values of these biomarkers did, however, not significantly differ between SR and AF patients (Supplementary Table S1).

Patients with newly diagnosed pAF had significantly higher age, larger aortic root and left atrial diameters, smaller end-systolic diameters, were less frequently smokers and had more frequently undergone a catheter ablation (accessory pathway or atrioventricular nodal reentry tachycardia ablation). Based on our previous study, threshold values of the developed 12-parameter ($L_{12} \ge 58.35$) and 4-parameter ($L_{4} \ge 63.32$) ECHO-AF scores that allowed prediction of pAF with 80% sensitivity were selected [17]. These score threshold values were applied in this study to prospectively evaluate the predictive performance of the developed predictive scores.

### Prospective validation of the ECHO-AF scores

**Fig. 2:**
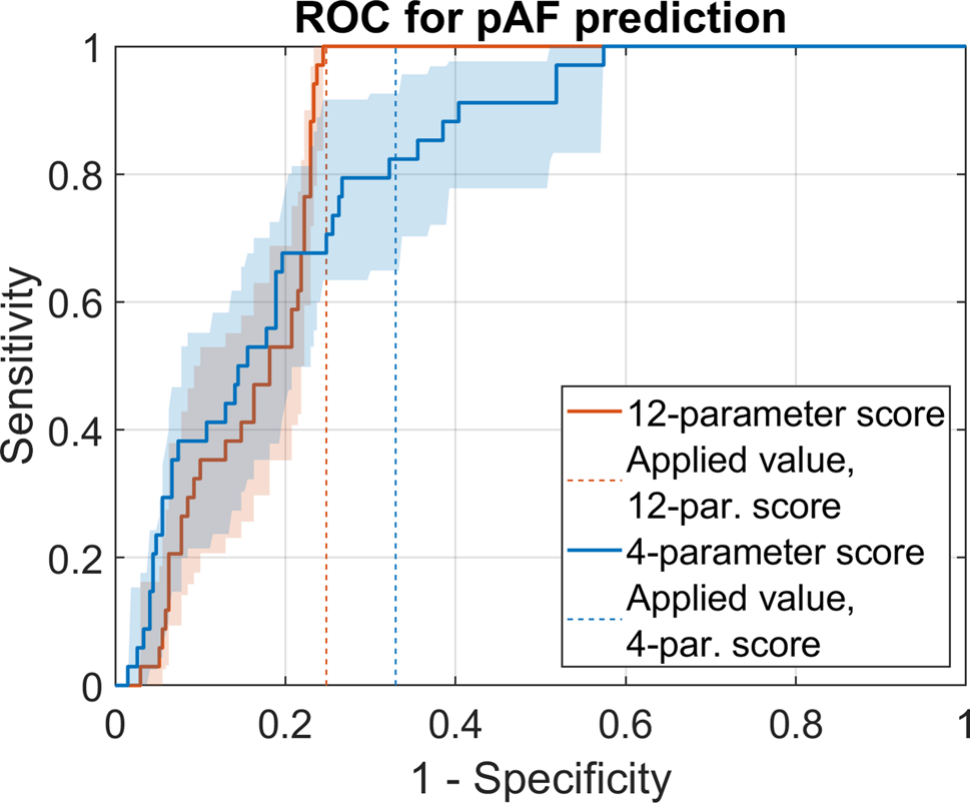
Performance of predictive scores for classification between pAF and SR. ROC curves were calculated for pAF prediction scores containing 12 parameters or 4 parameters (areas: 95% confidence intervals estimated by bootstrapping). Dashed lines indicate specificity values (75% for 12-parameter score, 67% for 4-parameter score) and sensitivity values (100% for 12-parameter score, and 82% for 4-parameter score) obtained by the pre-defined score thresholds

In this study, the 12-parameter and the 4-parameter scores showed sensitivities of 100% and 82% (95%-CI 65%, 93%), specificities of 75% (95%-CI 70%, 80%) and 67% (95%-CI: 61%, 73%), and areas under the receiver operating characteristic (ROC) curves of 0.84 (95%-CI: 0.80, 0.88) and 0.81 (95%-CI 0.74, 0.87) (Fig. 2). No confidence interval can be reported for the sensitivity of the 12-parameter score because all 34 pAF patients of a total of 305 patients were detected by its application, resulting a sensitivity of 100%. Values for sensitivity, specificity, and area under the ROC curve of 12-parameter and 4-parameter scores were higher than expected from the results of our previous retrospective study (Table 2) [17].

**Table 2:** Comparison between expected and observed performance of pAF prediction scores

	Results from this study		Values from retrospective study	
	12-Parameter score	4-Parameter score	12-Parameter score	4-Parameter score
Sensitivity	100%	82% (65%, 93%)	80% (73%, 87%)	80% (73%, 87%)
Specificity	75% (70%, 80%)	67% (61%, 73%)	68% (73%, 63%)	59% (65%, 52%)
AUC	0.84 (0.80, 0.88)	0.81 (0.74, 0.87)	0.80 (0.76, 0.84)	0.77 (0.72, 0.81)

In brackets, 95% confidence intervals are indicated (AUC, area under the ROC curve). For score development and selection of score thresholds for pAF prediction, confidence intervals were estimated by 100-fold cross-validation. In this study, these score thresholds were prospectively applied. Confidence intervals were estimated from bootstrapping with *n* = 1000 samples

The precision of the 12-parameter score, defined as the number of true positives divided by the sum of true and false positives, was 34% (95%-CI 25%, 44%). This value is comparable to the precision value of 36% that was previously estimated based on a retrospective dataset [17]. The 4-parameter score showed a precision of 24% (95%-CI 17%, 32%).

Taken together, the capabilities of the developed 12-parameter and 4-parameter ECHO-AF scores for pAF prediction could be validated indicating that the developed model scores represent a simple, highly sensitive and non-invasive tool for detecting pAF.

### Predictive performance of the ECHO-AF scores

**Fig. 3:**
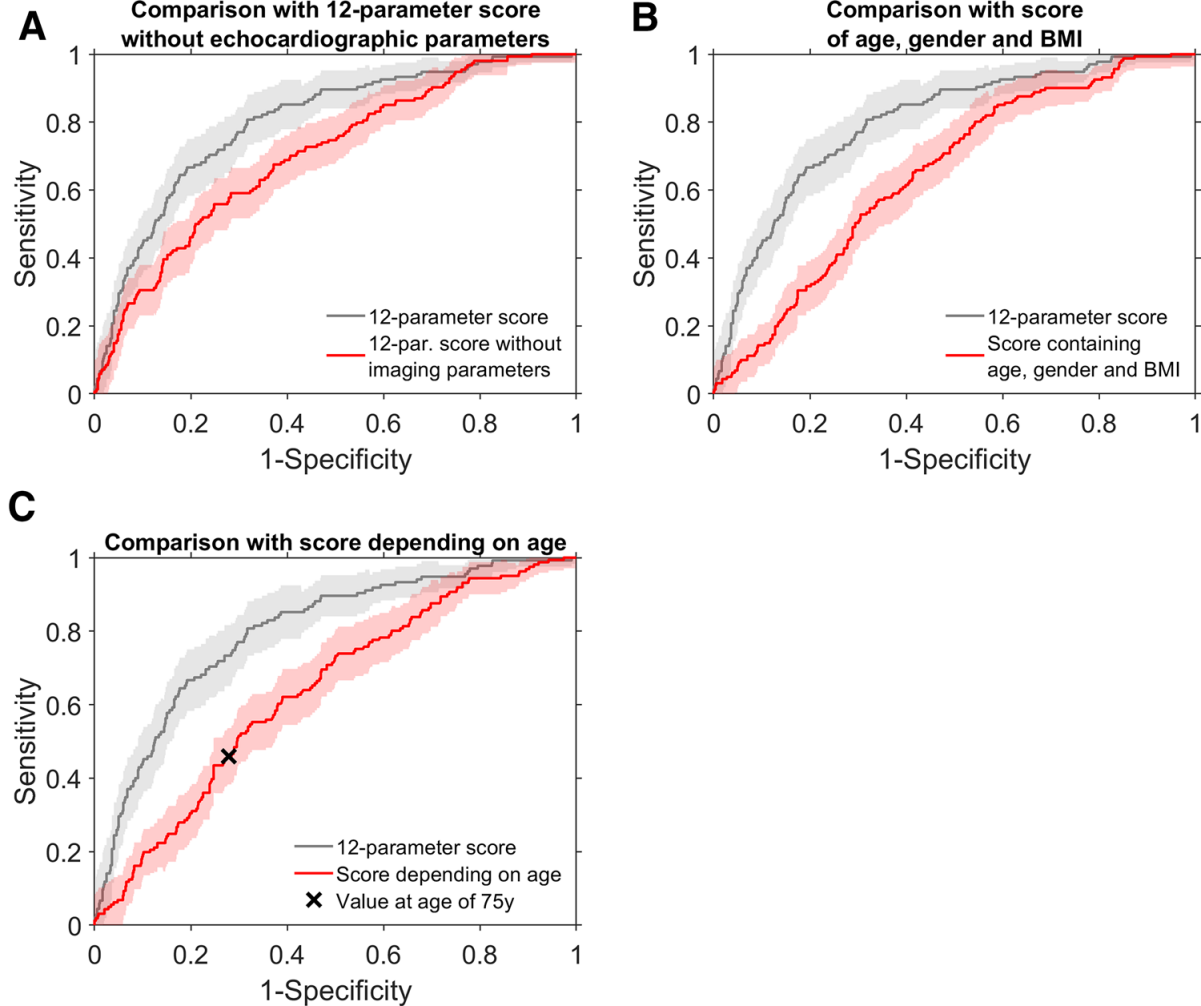
Comparison with ROC curves for reduced score variants: **a** the 12-parameter score without imaging parameters [AUC 0.70 (95%-C.I.: 0.65, 0.75); the original 12-parameter score with AUC 0.80 (95%-CI 0.76, 0.84) is indicated in grey], **b** a score for pAF prediction comprising the variables *age*, *gender* and *BMI* [AUC 0.65 (95%-CI 0.60, 0.69)], (C) a score for pAF prediction comprising only *age* as variable [AUC 0.64 (95%-CI 0.59, 0.69); the ‘ × ’ symbol indicates values of sensitivity and 1-specificity for a cut-off value of 75 years]

To put these results into perspective and assess the predictive value of echocardiographic imaging parameters, we retrospectively tested reduced and modified model variants based on the dataset that was originally used to derive the 12- and 4-parameter scores [17]. Excluding echocardiographic imaging parameters clearly reduced the predictive performance in the original dataset, comprising data from 1000 patients, indicated by an AUC value for the ROC curve of 0.70 (95%-CI 0.65, 0.75) compared to an AUC value of 0.80 (95%-CI 0.76, 0.84) for the full 12-parameter score (Fig. 3a). Other previous screening studies pointed out the predictive value of the parameters *age*, *gender* and *BMI* or were only based on the subject age [23, 25]. Accordingly, we tested logistic regression models reduced to these parameters. Whereas the model based on *age*, *gender* and *BMI* showed an AUC value under the ROC curve of 0.65 (95%-CI 0.60, 0.69; Fig. 3b), classification only based on age resulted in an AUC value of 0.64 (95%-CI 0.59, 0.69; Fig. 3c). For comparison, we further applied two previous scores (HAVOC and ACTEL) originally developed for detecting AF in patients after stroke or TIA that also resulted in ROC curves with comparably small AUC values (0.66 and 0.64; Supplementary Fig. S3A and B) [21, 22]. It was concluded that the predictive performance of the 12- and 4-parameter scores relies on echocardiographic parameters and that they should not be replaced by reduced variants.

### AF detection and characteristics of AF episodes

**Fig. 4:**
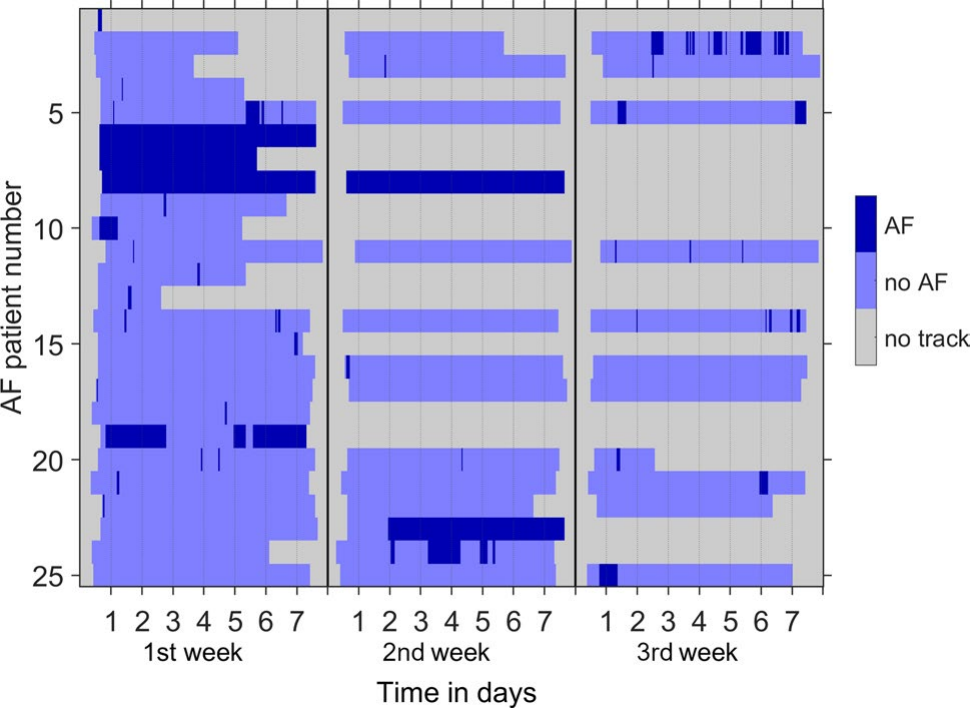
Temporal profiles of AF episodes. In 25 of a total of 34 patients with AF, up to 3 weeks of time-resolved ECG data were available (*y*-axis). Episodes are indicated by dark blue colour in ECG traces displayed parallel to the *x*-axis

To evaluate pAF score models, we collected extensive 3-lead Holter ECG recordings that are informative about characteristics of AF episodes in pAF patients. We characterized properties of AF episodes and analysed Holter ECG carrying durations that were necessary to detect pAF. In 25 of a total of 34 patients with AF, up to 3 weeks of time-resolved Holter ECG data were available (Fig. 4). In other pAF patients, pAF was detected by pacemaker devices or 12-lead ECG recorded after including a patient in the study. In several patients, AF was detected in the second or third week. Profiles of AF episodes strongly differed between AF patients. In 9 patients, only episodes of less than one hour were observed. In three patients, AF was persistent. We did not observe circadian rhythmicity of AF episode frequency (Supplementary Fig. S4). Score values were not significantly correlated with AF burden (Supplementary Fig. S5).

**Fig. 5:**
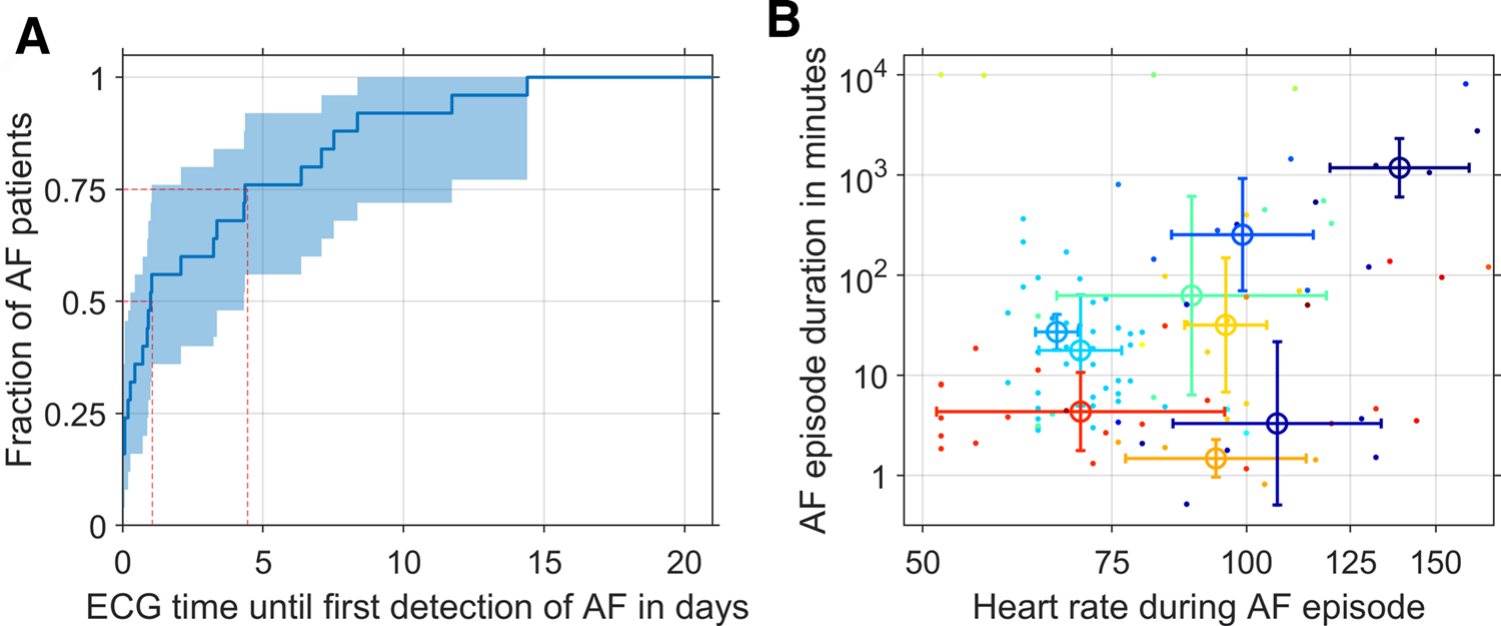
Characteristics of AF episodes. **a** Cumulative fractions of AF diagnoses for ECG monitoring durations (dashed red lines: ECG monitoring times until 50% or 75% AF diagnoses; shaded areas: 95% confidence intervals estimated by bootstrapping with *n* = 1000 samples). **b** AF episode durations and heart rates during AF episodes. Values of different patients are indicated by colours. Averages and standard deviation of log-scaled values are shown with error bars for all patients with more than three episodes (*n* = 9)

We evaluated how long ECG monitoring was necessary to detect AF in patients in case of our study. To this end, we extracted ECG monitoring times before the first AF episode and calculated cumulative fractions of AF diagnoses dependent on ECG monitoring times (Fig. 5a). Half of the AF patients were diagnosed within 1 day of ECG recording, whereas, 75% were diagnosed within 4.5 days ECG recording (dashed red lines in Fig. 5a). All pAF patients were diagnosed within 14.4 days of ECG monitoring. Figure 5b visualizes AF episode durations and heart rates during AF episodes for different patients. Interestingly, for patients with non-permanent AF (n = 22), heart rate in the presence of AF was weakly positively correlated with the AF episode duration (Spearman rank-order correlation *ρ* = 0.24, *p* = 0.014).

## Discussion

In this study, the capabilities of the developed 12-parameter and 4-parameter ECHO-AF scores for pAF prediction could be prospectively validated. In 34 study patients, pAF could be newly diagnosed. Further diagnostic validation and a positive CHA_2_DS_2_-VASc score confirmed that oral anticoagulation was necessary in these patients.

The moderate precision values (12-parameter score: 34%, 4-parameter score: 24%) result from the relatively small fraction of pAF patients (11%) relative to the fraction of SR patients in the study sample. Therefore, the scores cannot replace documentation of AF by ECG measurements but represent highly sensitive screening tests to select patients for further diagnostic validation of the presence of pAF by long-term Holter ECG measurements. Our investigation showed that the scores can be used to select patients, in which a long-term ECG monitoring should be conducted. For example, a positive score result could be coupled to further screening tools such as carrying a smart watch that can measure photoplethysmography signals or long-term Holter ECG monitoring. Using the scores to narrow the patient group, in which further AF screening is performed, can lead to a cost optimization [6].

Previously, several clinical studies defined AF prediction scores based on blood serum parameters [8–12, 23]. In contrast, our study focused on non-invasive clinical parameters, particularly on echocardiographic parameters. These are often available in cardiology practices, whereas, several serum biomarkers predictive for AF are frequently unavailable in patients not treated in a hospital setting. Furthermore, echocardiographic parameters reflecting measures of the cardiac anatomy, blood flow and tissue velocities are immediate indicators of the cardiac function. Therefore, it is likely that these are predictive for arrhythmias with pathophysiological consequences. Comparing the developed scores with variants without imaging parameters underlined that echocardiographic parameters are important for predicting the presence of AF.

We compared the ECHO-AF scores with the previously established HAVOC and ACTEL scores. ROC curves of these scores showed comparably small AUC values compared to the ECHO-AF scores. This discrepancy can be probably attributed to differences in patient collectives that were taken into account—the HAVOC and ACTEL scores were developed using clinical data of patients after stroke or TIA. In these patients, the prevalence of AF was substantially higher compared to our study population. As in case of the HAVOC and ACTEL scores, the C2HEST score represents an easy applicable tool for predicting AF by summing up integer numbers associated with the risk factors coronary artery disease, COPD, hypertension, age above 75 years, systolic heart failure and hyperthyroidism [26]. An internal validation of the score showed an AUC value of 0.75, whereas the external application showed an AUC value of 0.65, which is modest as compared to the ECHO-AF scores evaluated in this study. In general, scores calculated from summing up integer numbers associated with clinical features can be easily calculated but are less precise than scores represented by more complex equations, as in case of the ECHO-AF scores.

In contrast to the Apple Heart Study and the Huawei Heart Study, this screening trial was performed in a more selective patient cohort with higher pAF prevalence [4, 5]. In this context, it should be noted that the detection of an irregular rhythm by the algorithm used in a smart watch is not equivalent to the detection of AF, which has consequences for the false-positive detection rate of smart watches. It is important to note that rare arrhythmia episodes detected by a smart watch do not imply the same stroke risk as clinically diagnosed AF.

Other scores were developed to predict the 5-year or 10-year risk for developing pAF that require parameters measured by cardiac computed tomography or specialized laboratory tests (NT-proBNP, troponin T) [10, 12]. In contrast to these diagnostic procedures, echocardiography is routinely performed in many cardiological patients. Applying the developed pAF prediction scores in this patient population further increases the clinical value of echocardiographic parameters. Analysing cumulative fractions of AF diagnoses depending on the ECG monitoring duration indicated that an extended ECG monitoring for more than 1 week is required to reliably test for presence of pAF in accordance with previous long-term ECG monitoring studies [27, 28].

This study has limitations that have to be acknowledged. First, carrying durations strongly varied between subjects. Therefore, several cases of pAF might have been unrecognized. Our scores were evaluated in patients of different age groups, with different health profiles, and in hospital patients as well as in outpatient setting, which supports its general applicability. More selected subgroup analyses could improve the performance of the prediction tool. For example, subanalyses could be conducted to compare between hospital patients and outpatients, patients without or after thromboembolic events, or without and after heart surgical interventions. Additional parameters that were not included in the 47 parameters used to develop the pAF prediction scores, such as biomarkers (BNP, FGF-23, GDF-15), could further increase predictive performance [17, 23]. Validation of our developed pAF prediction scores in a larger study population would further reduce uncertainties of the parameters describing the predictive performance. An independent external prospective validation will be important for testing the general applicability of the ECHO-AF scores.

## Conclusion

The novel risk prediction scores adequately predicted pAF based on variables readily available during routine cardiac check-up and echocardiography. Thereby, the value of clinical parameters that are often known in cardiological patients can be increased and the early detection of pAF can be improved. Screening for pAF in patients undergoing echocardiography is reasonable because of a comparably high prevalence of pAF in this patient group. To simplify application of the scores, an online calculator is provided [20]. Collectively, the developed model scores represent a simple, highly sensitive and non-invasive tool for detecting pAF that can be easily implemented in clinical practice and might serve as a screening test to initiate further diagnostic investigations for documenting the presence of pAF.

## Electronic supplementary material

Below is the link to the electronic supplementary material.

Supplementary file1 (PDF 573 kb)
