# Supplementary material for: Prospective multicentric validation of a novel prediction model for paroxysmal atrial fibrillation
Source: Clin Res Cardiol. 2020 Nov 19;110(6):868–76. doi: 10.1007/s00392-020-01773-z (PMC8166666; doi:10.1007/s00392-020-01773-z)
Supplement: Supplementary file 1 — Supplementary file1 (PDF 573 kb) [file 392_2020_1773_MOESM1_ESM.pdf]

SUPPLEMENTARY MATERIAL

Prospective multicentric validation of a novel prediction model for paroxysmal atrial fibrillation

Constanze Schmidt[1,2], Sebastian Benda[1,2], Patricia Kraft[3], Felix Wiedmann[1,2], Sven Pleger[1,3], Antonius Büscher[1,2], Dierk Thomas[1,2], Rolf Wachter[4], Christian Schmid[5], Roland Eils[6,7], Hugo A. Katus[1,2], Stefan M. Kallenberger[6,7]

[1] Department of Cardiology, University of Heidelberg, Im Neuenheimer Feld 410, 69120 Heidelberg, Germany; [2] DZHK (German Center for Cardiovascular Research), Partner Site Heidelberg/Mannheim, University of Heidelberg, Im Neuenheimer Feld 410, 69120 Heidelberg, Germany; [3] Kardiologen am Brückenkopf, cardiology practice, Brückenkopfstraße 1/2, 69120 Heidelberg, Germany; [4] Clinic and Policlinic for Cardiology, University Hospital Leipzig, Liebigstraße 18, 04103 Leipzig, Germany and Clinic for Cardiology and Pneumology, University Medicine Göttingen, 37099 Göttingen, Germany;

[5] Department of Internal Medicine, GPR Klinikum Rüsselsheim, August-Bebel-Straße 59, 65428 Rüsselsheim am Main, Germany; [6] Digital Health Center, Berlin Institute of Health (BIH) and Charité, Anna-Louisa-Karsch-Straße 2, Berlin, 10178, Germany; [7] Division of Theoretical Bioinformatics, German Cancer Research Center (DKFZ), Im Neuenheimer Feld 267, Heidelberg, 69120, Germany

Corresponding author: Constanze Schmidt; University Hospital Heidelberg; Department of Cardiology, INF 410, 69120 Heidelberg, Germany; Constanze.Schmidt@med.uni-heidelberg.de

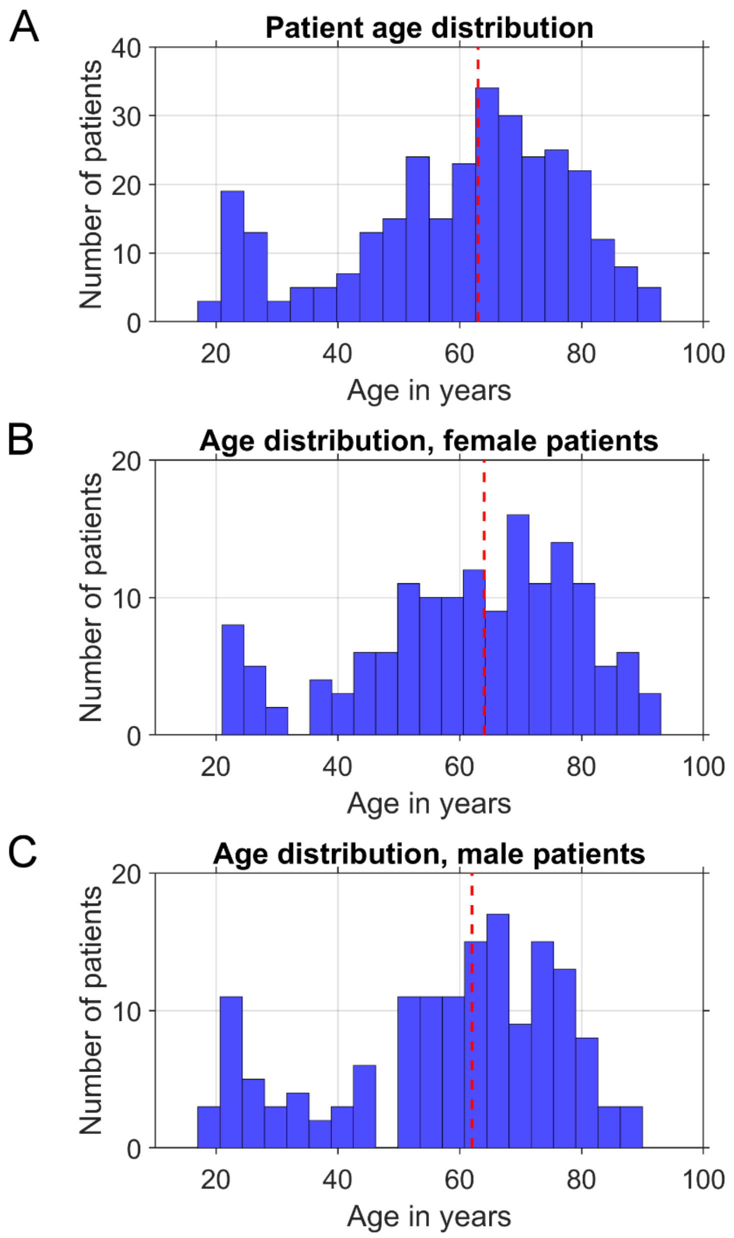

Fig. S1 Patient age distributions: patient age distributions are shown for (A) all patients, (B) female patients, and (C) male patients. Median ages are indicated by dashed red lines (median age: 63, median age in female patients: 64, median age in male patients: 62)

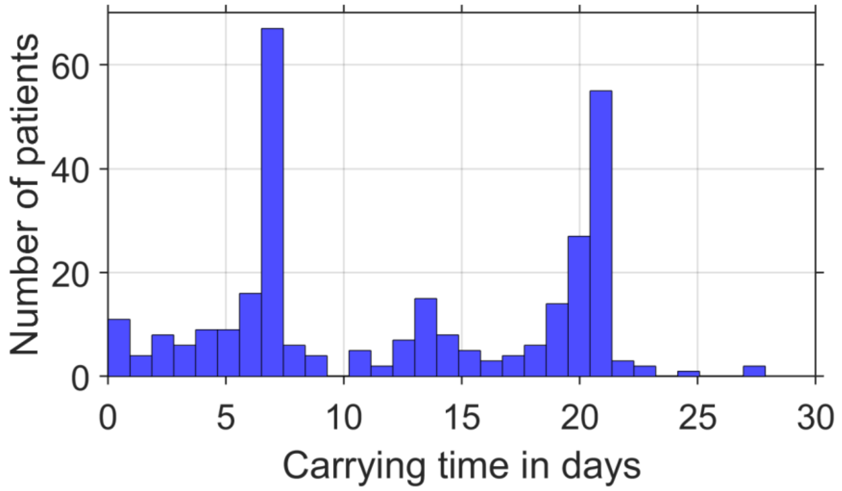

Fig. S2 Distribution of Holter ECG device carrying times. Holter ECG devices were carried in intervals of one week length for up to three weeks, which explains histogram peaks around 1 week, 2 weeks and 3 weeks. A few patients carried the device for four weeks

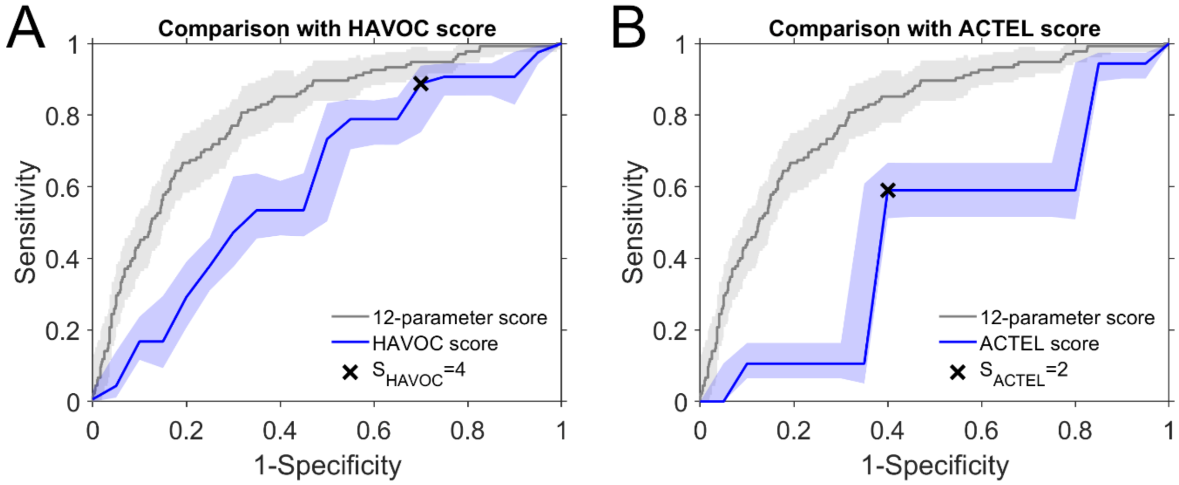

Fig. S3 Comparison with ROC curves for other prediction scores: (A) the HAVOC score [AUC 0.66 (95%-C.I.: 0.61, 0.70); the '×' symbol indicates sensitivity and 1-specificity values for the suggested cut-off score value of 4], and (B) the ACTEL score [AUC 0.64 (95%-C.I.: 0.60, 0.68); the '×' symbol indicates sensitivity and 1-specificity values for the suggested cut-off score value of 2]

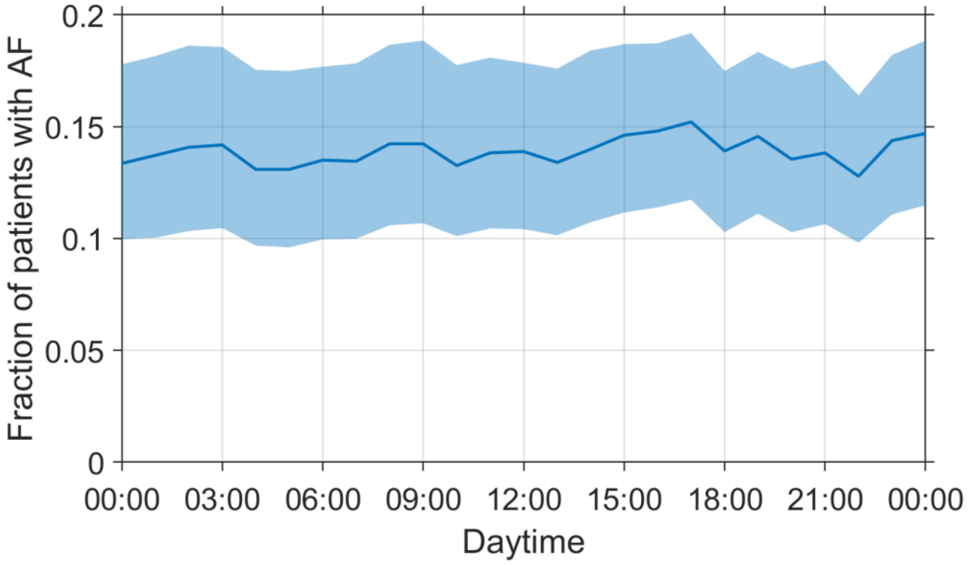

Fig. S4 Circadian frequency of AF episodes: for all patients and all days, fractions of AF presence over daytime were calculated (shaded areas: 95% confidence intervals estimated by bootstrapping with n=1000 samples)

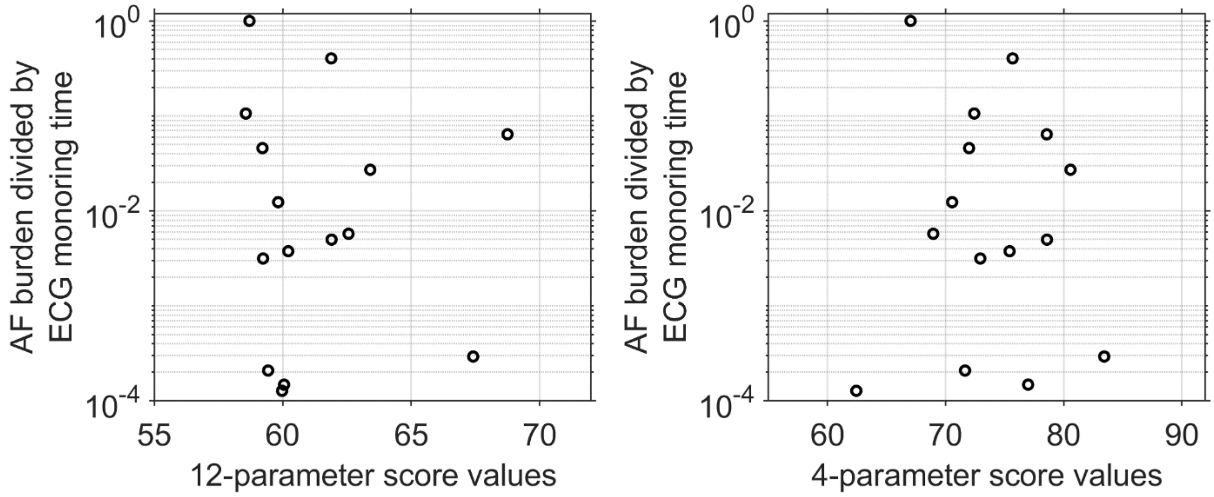

Fig. S5 Values of pAF scores and AF burden normalized by ECG monitoring time. Values of the 12-parameter score (left) and the 4-parameter score were not correlated with AF burden

Table S1. Comparison of serum parameters between patient groups.

	**SR**	**pAF**
Parameters		
GFR (CKD-EPI), ml/(min·1.73m²)	83.8±16.4	74.3±22.1
	(n=34)	(n=33)
Serum creatinine, mg/dl	0.86±0.18	1.11±0.99
	(n=35)	(n=30)
NT-proBNP, ng/l	4,458±8,375	1,821±3,959
	(n=14)	(n=22)
Troponin T hs, ng/l	190±573	159±359
	(n=23)	(n=26)

Means, standard deviations and numbers of patients are indicated. GFR (CKD-EPI), glomerular filtration rate estimated by the chronic kidney disease epidemiology collaboration formula; NT-proBNP, N-terminal pro-B-type natriuretic peptide; hs, high-sensitive. Differences between SR and pAF groups, assessed by ANOVA, were not significant.
